# The “Timeline Debriefing Tool”: a tool for structuring the debriefing description phase

**DOI:** 10.1186/s41077-019-0119-4

**Published:** 2019-12-19

**Authors:** Thierry Secheresse, Séverine Nonglaton

**Affiliations:** 1 CEnSIM Healthcare Simulation Center, Metropole Savoie Hospital, BP 31125, 73011 Chambéry Cedex, France; 2 Laboratory of Research on Acquisition in Context (LaRAC), University Grenoble Alpes, CS 40700, 38058 Grenoble Cedex 9, France

**Keywords:** Simulation, Debriefing, Description phase, Structured debriefing, Debriefing method

## Abstract

Several recent literature reviews have been published with the aim to determine how to optimise a debriefing. A main element found in these reviews was the importance of structuring the debriefing. Within the steps usually outlined in the debriefing, the description phase allows participants to describe their recollections and establish a shared mental model of what happened during the simulation. The description phase is used in many debriefing models but how to realise this description remains unclear. We provide an original tool to ensure a highly structured description phase: the “Timeline Debriefing Tool”.

The Timeline Debriefing Tool, or TDT, is constructed on visual support such as a whiteboard or a flipchart. It allows for a clear description phase, makes the process more dynamic, promotes exchanges between participants and establishes a clear and shared vision of the simulation in visual support which can be used by the instructor in the analysis phase. Moreover, the timeline allows participants to discover their performance gaps by themselves, thus beginning deeper cognitive processing in the participants’ mind and promoting reflection in the analysis phase.

Debriefing is said to be a core element of simulation-based learning [1–4]. Several recent literature reviews were published with the aim to determine how to optimise debriefings and maximise learning during simulation-based healthcare education [5–11]. The main results found in these reviews can be summarised in five elements: (1) the importance of debriefing in the learning process, (2) the non-superiority of using video in debriefing, (3) the multiplicity of the debriefing methodologies, (4) the reflective approach used in the debriefing and (5) the importance to structured debriefing. Using a structured debriefing framework appears to be a core element to achieving positive learning outcomes [9, 12]. Four steps are usually described in the debriefing [13].

- A reaction phase to express the participants’ feelings, decrease the emotional stress and allow expression of the participants’ agendas.
- A description phase to recall what happened during the simulation.
- An analysis phase to explore the participants’ frame of mind from the results and actions observable in the simulation in a reflective discussion about the internal processes that guided their actions [14].
- A summary phase to highlight the take-home messages and plans for future actions.

Several debriefing methodologies have been described using these different phases, ranging from three-phase to multi-phase structures [15]. The description phase is used in many debriefing models but how to realise this remains unclear. Based on the authors’ experience in faculty development for simulation debriefings, we describe a highly structured strategy that we have found helpful for organising the description phase: the “Timeline Debriefing Tool”.

## The “Timeline Debriefing Tool”: how to use it?

The Timeline Debriefing Tool is used during the description phase using visual support such as whiteboard or flipchart and unfolds in the following steps.

1. The debriefer stands in front of the board and draws a horizontal arrow on the right edge of the board and then asks the participants to describe the status of the simulated patient at the end of the scenario. The debriefer then notes the main characteristics of the patient under the arrow as described by the participants. The debriefer can supplement points that might be missing.
2. The debriefer places himself on the left edge of the board and draws a vertical bar and asks the participants how the simulated patient was at the beginning of the scenario. The debriefer notes the main elements under the vertical bar in interaction with the participants as described under 1.
3. The debriefer draws a horizontal line joining the centre of the vertical bar to the arrow and invites all the participants to come to the board. Then the debriefer asks the participants to write on the timeline all that happened during the simulation. The debriefer specifies that, during this description phase, only factual elements are required, without analysis.

Three precise instructions are delivered:

- An outline of the importance of the participants’ role in this description: “We do not know what went through your mind during the scenario. We would like to know how you experienced the situation during the scenario*.*”
- In-depth reporting: the debriefer encourages the participants to report every detail, regardless of how peripheral it may seem to the main elements. This deep recall instruction is important for two reasons. First, the participants may only initially report information they assume to be important. Thus, deep recall instruction supports participants in reporting details that they think might be irrelevant but which could prove to be highly important within the framework of the debriefing. Second, recalling partial details may lead to subsequent recall of relevant information as details of minor importance tend to act as powerful retrieval cues that allow participants to gain access to their memories with greater ease. During a specific situation, individuals memorise the most important information but also a vast amount of information about the environment and their physical and mental states. This contextual information helps in the retrieval of critical information and the quality of the recall increases in proportion to the number of contextual elements present during encoding time and used during the recalling phase [16, 17]. We formulate this instruction like this: “Try to write everything down that comes to your mind to be as complete as possible. Everything that comes to your mind can be interesting. Do not hesitate to share details, however small or irrelevant. Think about everything you have seen and done during the case. Take into account every detail, regardless of how small or insignificant it may seem to you.”
- A Mental Reinstatement of Environmental and Personal Contexts instruction: the participants are asked to mentally revisit the scenario. According to the encoding specificity theory, recall is enhanced when the cues have some degree of similarity to cues that were present at the time of encoding. With this mental recontextualization instruction, the aim of the debriefer is to get a better recollection of the scenario [18]. “Try to mentally get back into the situation, to relive that simulation in your head. Think about where the simulation took place. What you have heard or done. What did the other people do with you for the patient?”

4. While the participants exchange and write the sequence of actions on the board, the debriefer supports the participants’ description using open questions and non-verbal behaviours. The debriefer keeps them focused on the factual description of the scenario without analysing the internal processes that guided the participants’ actions.
5. Once the participants have finished describing the events, the debriefer asks if they have other things to add. If participants who observed the simulation are present, they can add information and help complete the timeline.
6. Then the debriefer can add precise and objective data on the timeline such as the duration of the main events or the time when they happened. These details are added in a factual approach. (e.g. “We can specify when the main elements were performed. So intubation was performed at 3.12 min and first shock at 4.28 min”). These elements are written on the timeline in a different colour (green, in our example).
7. Then the debriefer asks the participants whether this representation corresponds to the common vision of the situation and proposes to move on to the analysis phase.

**Fig. 1:**
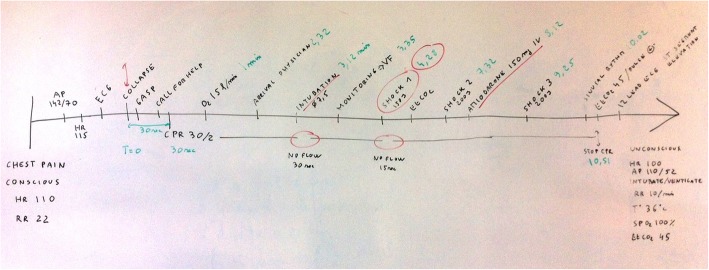
Example of a cardiac arrest scenario timeline

In the example shown in Fig. 1, a whiteboard was used to construct the timeline. Other supports could be used such as flipchart, large piece of paper, post-its or even physical representation, e.g. a rope on the floor.

## Discussion

The description phase is an important step of the debriefing process with two main objectives: detail the participants’ recollection of the simulation, and establish a shared mental model of what happened during the event [19, 20]. Moreover, the description phase allows preparing the analysis phase in highlighting the main points of interest that will be explored in the analysis phase in an active, reflective and learner-centred approach [21].

Performing the description phase using the Timeline Debriefing Tool reinforces the building of a structured process with very clear information, preparing us for the analysis phase. In a four-phase debriefing model (i.e. reactions, description, analysis, summary), using the Timeline Debriefing Tool during the description phase takes about 25% of the entire debriefing duration. It makes the debriefing more dynamic, with all participants involved in building the timeline, thus promoting productive exchanges. This tool allows the structuring of the description phase in a chronologic approach during the first part of the debriefing. It provides a visual representation, established by the participants in interaction with the debriefer, of the key issues and main steps in relation to the learning goals. Visual representation is used in many clinical education strategies to present and structure information [22, 23]. At the end of the description phase, the timeline establishes a clear and shared vision of the simulation for all the participants that can be used by the debriefer in the analysis phase. In this way, in the analysis part of the debriefing, the debriefer can move from a chronologic approach to specific topics and a learner-centred approach [24].

The Timeline Debriefing Tool allows highlighting the topics that will be covered in the analysis phase. The debriefer can specify particular elements to debrief surrounding them in a different colour on the timeline (for example, intubation before electric shock, no flow or wrong dosage of amiodarone in red colour in our example): “I would like to discuss intubation, electric shock, no flow …” In addition, the debriefer can also ask the participants if they have a special topic to debrief on, in relation to the learning goals, and write it on the board. So, at the end of the description phase, participants have a shared mental model of what happened during the scenario and know exactly which topics will be discussed. The analysis phase can then be structured in a topic-oriented approach, using for each learning objective, the appropriate strategy such as learners self-assessment, Socratic questions or focused facilitation [25–29].

Another value of this debriefing tool is to allow participants to discover their own performance gaps by themselves while they write the sequence of actions on the board. Thus they can begin to reflect immediately that can lead to a process of cognitive conflict in the participants’ minds. According to constructivism, learning needs cognitive conflict that is to say conflict between learners’ prior knowledge and new contents to be taught [30]. In simulation-based education, cognitive conflict could arise when participants’ knowledge does not allows them to resolve a new situation or when there is a difference between the participants’ knowledge and behaviour during the scenario. One key element will be to make the cognitive conflict meaningful for learners [31]. The visual support created by the participants on the Timeline Debriefing Tool may make implicit things explicit as highlighting the gap between what the participants wanted to do and what they actually did. For example, in a cardiac arrest scenario, while writing the sequence of actions on the timeline, the participants can realise that they performed the initial defibrillation too late, “we made the first defibrillation at this moment while we should have done it sooner”. The debriefer can resume this element during the analysis phase using the visual support of the timeline and help the participants to reflect on a “good judgement approach” [32, 33]: “you wrote you did the first defibrillation at this moment and said you should have done it sooner. We can see that it takes 2 minutes to make the first defibrillation. Can you explain why?” Thus, the timeline description can be used to promote reflection during the analysis phase [34].

In a recent publication, Fraser et al. discussed the multiple competing priorities for the debriefer attention that can contribute to a high cognitive load [35]. According to Cognitive Load Theory [36], these potential cognitive loads could adversely affect debriefer performance and consequently participants’ outcomes. Using the Timeline Debriefing Tool could optimise the debriefer cognitive load during the description phase in structuring the debriefing. Furthermore, the visual representation of what happened in the scenario and the visual of the learning objectives may focus both the debriefer and participants’ attention about the main points of interest during the debriefing and promote appropriate cognitive load [37].

## Limitations and future directions

The Timeline Debriefing Tool is used routinely in our simulation centre and is taught in the faculty development. From the debriefer’s perspective, this tool is easy to use, clarifies what happened during the scenario and allows structure. From the participants’ perspective, the Timeline Debriefing Tool structures the debriefing in an organised way. The debriefing progresses logically rather than jumping around from point to point. The written timeline provides concrete examples and feedback to get participants to think about their performance and provokes in-depth discussion.

From our perspective, how to use the Timeline Debriefing Tool needs to be trained. It is not just about writing a timeline on a board. Precise instructions need to be given to the participants to promote active participation and exchanges. Moreover, the debriefer must keep in mind the different phases of the debriefing, and especially, the importance of the analysis phase. Thus, the debriefer has to ensure that the description phase with the Timeline Debriefing Tool does not take too long to complete, in order to allow enough time for the analysis and summary phases. In other words, the debriefer is the “time keeper” of the debriefing.

From our experience, the Timeline Debriefing Tool seems well suited for healthcare and technical simulation scenarios such as emergency situations or anaesthesia procedure simulations. For scenarios such as relational simulations (e.g. breaking bad news or care-related-damage-disclosure scenarios), the Timeline Debriefing Tool seems less suitable due to the characteristics of the educational objectives. However, future studies would be helpful to determine in which context and for which type of educational objectives the Timeline Debriefing Tool is best suited.

The Timeline Debriefing Tool presented here is based on our collective experience of debriefing and faculty development and we acknowledge the lack of empirical evidence to describe the best use of this debriefing strategy. However, our experience in using this tool for 2 years and the importance of positive feedbacks both from the participants and the faculty debriefers lead us to share this tool with the simulation community. We encourage faculty debriefers to try the Timeline Debriefing Tool while future studies will better specify its use cases.

## Data Availability

Data and materials are available from: Thierry Secheresse, MD CEnSIM Healthcare Simulation Center Metropole Savoie Hospital BP 31125 73011 Chambéry Cedex (France) Email: thierry.secheresse@ch-metropole-savoie.fr
